# The complete chloroplast genome sequence of the *Cucurbita ficifolia* Bouché (Cucurbitaceae)

**DOI:** 10.1080/23802359.2021.1981171

**Published:** 2021-09-30

**Authors:** Zhou Cong, Lijuan Cai, Yu Zhang, Wenzhen Su, Huiying Li, Qianglong Zhu

**Affiliations:** a Department of Horticulture, College of Agronomy, Jiangxi Agricultural University, Nanchang, P.R. China; b Nanchang Business College, Jiangxi Agricultural University, Nanchang, P.R. China

**Keywords:** *Cucurbita ficifolia* Bouché, chloroplast genome, fig-leaf gourd

## Abstract

*Cucurbita ficifolia* Bouché is an important germplasm resource used for rootstock and hypoglycemic food in Cucurbitaceae. The complete chloroplast genome sequence of *C. ficifolia* has been determined in this study. The total genome size is 157,533 bp in length and contains a pair of inverted repeats (IRs) of 25,639 bp, which were separated by large single copy (LSC) and small single copy (SSC) of 88,112 bp and 18,143 bp, respectively. A total of 130 genes were predicted including 86 protein-coding genes, eight rRNA genes and 36 tRNA genes. Further, Maximum-likelihood phylogenetic analysis revealed that *C. ficifolia* is a base clade of genus Cucurbita and closer to Cucurbita maxima. The chloroplast genome of *C. ficifolia* would promote the germplasm exploration, phylogenetic relationships, and molecular biology researches in Cucurbita.

*Cucurbita ficifolia* Bouché. commonly known as ‘fig-leaf gourd’, is an important germplasm resource with strong resistance to drought, salinity, coldness and soil-borne diseases, therefore, it is worldwide used for rootstock to improve the plant tolerance to abiotic and biotic stress in Cucurbitaceae (Yuan et al. 2010; Ding et al. 2019). The fruit of *C. ficifolia* contains D-chiro-inositol with the hypoglycemic activity in man and other animals (Xia and Wang 2006), is commonly used as a vegetable insulin for remedying diabetes in Asia, Africa and South America (Moya-Hernández et al. 2020), and it seeds have a high content of unsaturated fatty acids, which enables their use as a good and healthy oil to be used in the food industry (Carrillo et al. 2018). *C. ficifolia* was origin from the high-altitude localities in Central and South America and now distributed all over the world. *C. ficifolia* is belong to the Cucurbitaceae (cucurbit) family and consist of at least five domesticated and more than ten wild species (Sun et al. 2017), it is difficult to characterize and identify diverse Cucurbita germplasm by traditionally morphological characters, which hindering the excavation and utilization of *C. ficifolia*. Chloroplast genome is a molecular resource for developing DNA markers for identification and domestication of Cucurbita (Logan et al. 2015). However, to the best of our knowledge, there are no reports that the chloroplast genome of *C. ficifolia* was taken as a molecular resource. Thus, the goal of this study is to sequence the chloroplast complete genome of *C. ficifolia* with the hope to promoting the studies on the germplasm exploration, phylogenetic relationships, and molecular biology researches.

The *C. ficifolia* was collected at Jinggangshan, Jian, Jiangxi (114°97′E, 27°12′N) in October 2020, and the seeds was deposited and planted at the horticultural teaching station of Jiangxi Agricultural University under the voucher number: hz23. The genomic DNA was extracted from fresh and healthy leaves of *C. ficifolia* using the modified CTAB method (Porebski et al. 1997). The high-quality DNA was sent to company and used for library construction and genome sequencing on the BGISEQ-500 (BGI, Shenzhen, China). After sequencing and base quality control, about 2 Gb of clean sequence data in fastq format was obtained. The draft chloroplast genome sequence was assembled by using the Plasmidspades.py in SPAdes 3.15.3 (Bankevich et al. 2012). Contigs representing the chloroplast genome were retrieved, ordered, and incorporated into a long scaffold by aligning to the chloroplast genome of *Cucurbita maxima* (NC 036505.1) using SAMtools and BlastN. The gaps in the scaffold were closed using GapCloser v1.12-r6, and long sequence without gaps was mapped by pair-end reads to validate its completeness. Finally, the long sequence was taken as complete genome sequence and annotated using CPGAVAS2 and GeSeq (Tillich et al. 2017; Shi et al. 2019), the problems in annotations was corrected by Sequin.

The complete chloroplast genome of *C. ficifolia* was submitted to GenBank with accession: MZ578000, the length is 157,533 bp with 36.22% GC contents, and it contains a pair of IRs (25, 639 bp) detached by the LSC (88,112 bp) and SSC (18,143 bp) regions, to exhibit a typical quadripartite structure. There is a total of 131 genes, including 86 protein-coding genes, eight rRNA genes and 36 tRNA genes; six of the protein-coding genes, six of the tRNA genes and four rRNA genes are duplicated within the IRs.

To determine the phylogenetic position of *C. ficifolia*, a phylogenetic relationship was analyzed on the complete chloroplast genome of C. ficifolia and other 17 species in Cucurbitaceae with Maximum-likelihood (ML) method using the program MAFFT v7.407 (Nakamura et al. 2018) and MEGA v10.0.4 (Kumar et al. 2018). The phylogenetic tree showed that *C. ficifolia* is a base clade of genus Cucurbita and closer to *C. maxima* (Figure 1), the conclusions further support the previous research results (Chomicki et al. 2020).

**Figure 1.**
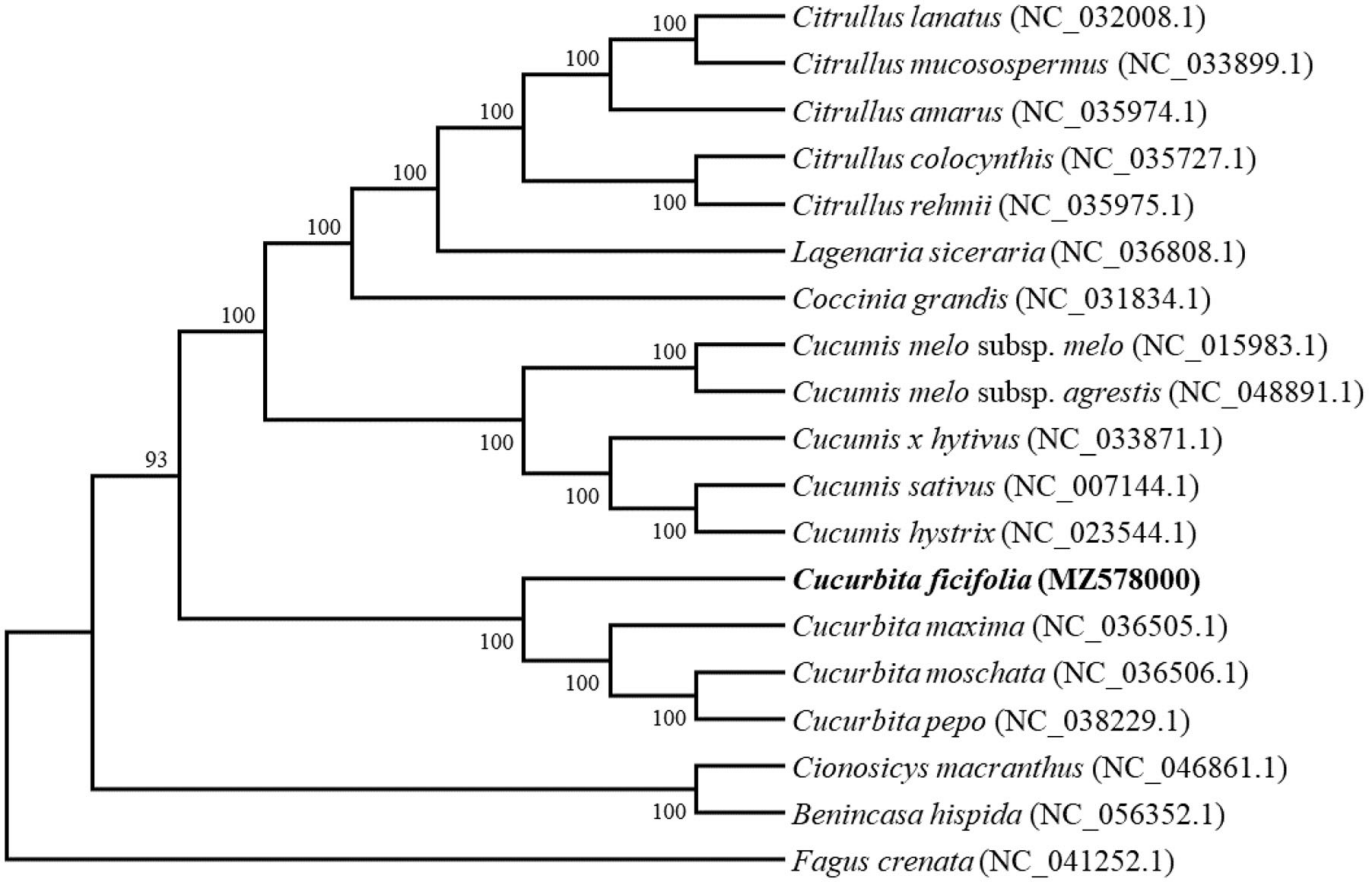
Phylogenetic tree showing relationship between *Cucurbita ficifolia* and other 17 species in Cucurbitaceae, Fagus crenata (NC_041252.1) was taken as the outgroup. Phylogenetic tree was constructed based on the complete chloroplast genomes using maximum-likelihood (ML) with 1000 bootstrap replicates. Numbers in each the node indicated the bootstrap support values.

## Data Availability

The genome sequence data that support the findings of this study are openly available in GenBank of NCBI at https://www.ncbi.nlm.nih.gov/nuccore/MZ578000.1/ under the accession no. MZ578000.
